# The critical role of ERK in death resistance and invasiveness of hypoxia-selected glioblastoma cells

**DOI:** 10.1186/1471-2407-9-27

**Published:** 2009-01-23

**Authors:** Jee-Youn Kim, Yong-Jun Kim, Sun Lee, Jae-Hoon Park

**Affiliations:** 1 Brain Korea 21 Project Center, College of Medicine, Kyung Hee University, Seoul 130-701, Korea; 2 Department of Pathology and Medical Science and Engineering Research Center for Bioreaction to Reactive Oxygen Species, Kyung Hee University, Seoul, South Korea

## Abstract

**Background:**

The rapid growth of tumor parenchyma leads to chronic hypoxia that can result in the selection of cancer cells with a more aggressive behavior and death-resistant potential to survive and proliferate. Thus, identifying the key molecules and molecular mechanisms responsible for the phenotypic changes associated with chronic hypoxia has valuable implications for the development of a therapeutic modality. The aim of this study was to identify the molecular basis of the phenotypic changes triggered by chronic repeated hypoxia.

**Methods:**

Hypoxia-resistant T98G (HRT98G) cells were selected by repeated exposure to hypoxia and reoxygenation. Cell death rate was determined by the trypan blue exclusion method and protein expression levels were examined by western blot analysis. The invasive phenotype of the tumor cells was determined by the Matrigel invasion assay. Immunohistochemistry was performed to analyze the expression of proteins in the brain tumor samples. The Student T-test and Pearson Chi-Square test was used for statistical analyses.

**Results:**

We demonstrate that chronic repeated hypoxic exposures cause T98G cells to survive low oxygen tension. As compared with parent cells, hypoxia-selected T98G cells not only express higher levels of anti-apoptotic proteins such as Bcl-2, Bcl-X_L_, and phosphorylated ERK, but they also have a more invasive potential in Matrigel invasion chambers. Activation or suppression of ERK pathways with a specific activator or inhibitor, respectively, indicates that ERK is a key molecule responsible for death resistance under hypoxic conditions and a more invasive phenotype. Finally, we show that the activation of ERK is more prominent in malignant glioblastomas exposed to hypoxia than in low grade astrocytic glial tumors.

**Conclusion:**

Our study suggests that activation of ERK plays a pivotal role in death resistance under chronic hypoxia and phenotypic changes related to the invasive phenotype of HRT98G cells compared to parent cells.

## Background

Adequate supplies of oxygen and nutrients from the vascular network are requisite for robust tumor growth. However, uncoordinated growth rates between the tumor parenchyma and the vascular connective tissue expose cancer cells to a hypoxic environment, thereby limiting further growth of the tumor mass. Conversely, hypoxia may select for cancer cells with an aggressive behavior because tumor cells that can overcome the unfavorable oxygen conditions will survive and proliferate [1-3]. Hypoxic selection may result in a poor response to treatment, recurrence of cancer, and metastasis. Therefore, investigation of the phenotypic changes induced by chronic hypoxia and the underlying molecular mechanisms is fundamental to develop appropriate and effective cancer treatment modalities as well as to comprehend tumor biology.

Cellular stresses such as hypoxia induce activation of diverse signaling pathways, which allow cells to survive in unfavorable conditions. Among the activated signaling pathways, mitogen-activated protein kinases (MAPKs) are early responders to hypoxic conditions [4]. MAPKs are serine/threonine kinases that regulate various cellular responses such as proliferation, differentiation, and apoptosis [4,5]. The extracellular signal-regulated kinase, ERK, a subfamily member of MAPKs, is a key molecule responsible for survival under hypoxia [6,7]. ERKs induce hypoxia inducible factor-1 (HIF-1), a master transactivator in hypoxic conditions, which in turn regulates transcription of hypoxia-adaptive proteins such as VEGF, erythropoietin, and Glut-1 [8-10]. However, while some candidate proteins responsible for adaptation in hypoxia are well characterized, the identity of proteins involved in chronic hypoxia selection and death resistance are largely unidentified.

This study was designed to identify the molecular basis of phenotypic changes triggered by chronic hypoxia. By establishing death-resistant cells selected by repeated episodes of exposing the T98G glioblastoma cell line (HRT98G) to hypoxia and reoxygenation, we found that ERK plays a pivotal role in hypoxia selection and resistance. In addition, we show that high expression of phosphorylated ERK (p-ERK) is responsible for HRT98G cells having a more invasive phenotype than the parent cells. Together, our results suggest that ERK is a key molecule involved in death resistance to chronic hypoxia.

## Methods

### Cell culture, hypoxic conditions, and cell death assay

The T98G glioblastoma cell line was obtained from the American Type Culture Collection (Rockville, MD, USA) and cultured in Dulbecco's modified Eagle's (DMEM) supplemented with 10% fetal bovine serum. For hypoxic condition, cells in a degassed medium were exposed to 0.5% O_2 _balanced with 5% CO_2_/94.5% N_2 _in a hypoxic chamber (In vivo2, Ruskinn, UK), followed by incubation in normal culture conditions for recovery. The cell death rate was determined by the trypan blue exclusion method.

### Antibodies and reagents

Antibodies used in this study were obtained from Cell Signaling Technology (Beverley, MA, USA). Anti-HGTD-P antibody was produced as previously described [11]. All reagents were purchased from Sigma-Aldrich, Inc. (St. Louis, MO, USA), unless otherwise specified.

### Immunoblot analysis

Cells were harvested and suspended in lysis buffer containing 10 mM Tris-HCl (pH 7.4), 1% NP-40, 0.1% sodium deoxycholate, 0.1% SDS, 150 mM NaCl, 1 mM EDTA, 1 mM EGTA, 0.5 mM phenylmethylsulfonyl fluoride, 1.1 mM Na_3_VO_4_, and 10 mM NaF. Extracted proteins were separated by SDS-PAGE on 12% polyacrylamide gels and electrophoretically transferred onto nitrocellulose membranes. Membranes were probed with primary antibody, and then incubated with horseradish peroxidase-coupled secondary antibody. Detection was performed with a chemiluminescence-based detection kit (Amersham Pharmacia Biotech., UK).

### Ras activity assay and determination of reactive oxygen species (ROS)

Ras activity was measured with a Ras activity assay kit (Upstate, MA, USA) detecting Ras bound to the Ras-binding domain of Raf-1, as described previously [12]. For determination of ROS, cells were suspended in PBS containing 5 μM dichlorofluorescine diacetate (DCFH-DA), followed by incubation at 37°C for 30 min. After washing with PBS, cells were analyzed by cytomics FC500 using CXP software (Beckman Coulter, CA, USA).

### Knockdown of ERK

HRT98G cells were transfected with siRNA specific to ERK (siERK) (Santa Cruz Biotech., CA) or control siRNA (siControl) according to manufacturer's protocol. Knockdown of the target gene was confirmed by immunoblotting.

### Invasion assay

Invasion assays were carried out using the cell invasion kit (Chemicon, USA) according to the manufacturer's protocol. Briefly, 10^4 ^cells were plated on a Matrigel-coated transwell invasion chamber with or without PD98059 or phorbol myristate acetate (PMA), and incubated at 37°C for 24 h. Non-invading cells were removed by wiping the upper side of the membrane of the transwell. Invading cells were fixed with methanol and stained with hematoxylin. Three independent invasion assays in triplicate were performed. On average, six random fields were counted under a light microscope.

### Tumor samples

The study was approved by the institutional review board of Kyung-Hee University Hospital. Twenty cases of astrocytic glial tumor samples [7, World Health Organization (WHO) grade I; 3, grade II; 3, grade III; 7, grade IV] were obtained from the hospital. Tissue samples were fixed with 10% phosphate-buffered formalin, embedded in paraffin, and sectioned into sections of 4 μm thickness.

### Immunohistochemistry

Formalin-fixed, paraffin-embedded tissue sections were deparaffinized, rehydrated, and washed twice for 5 minutes in wash buffer (50 mM Tris/HCl, pH 7.6, 50 mM NaCl). Endogenous peroxidase was quenched with peroxidase blocking solution (Dako, Carpinteria, CA) for 10 minutes. Slides were washed as before and then incubated in blocking solution (Dako) for 1 hour. This was followed by incubation with anti-p-ERK, (Cell Signaling Technology) or anti-HGTD-P rabbit polyclonal antibody for 1 hour. The slides were washed twice and further incubated with biotinylated secondary antibody and avidin-conjugated horseradish peroxidase. The slides were visualized using the DAB substrate-chromogen system (Dako) and counterstained with hematoxylin. Evaluation of immunohistochemical staining was performed by arbitrary quantitative scoring system. Fields with no positively-stained cells were scored as 0. Fields with less than 25% positively-stained cells were scored as 1; fields with between 26 to 50% positively-stained cells were scored as 2; fields with positively-stained areas between 51 to 75% were scored as 3; and fields with a positively-stained area greater than 76% were scored as 4. For each case, the mean score (sum of scores for each field/fields counted) was calculated.

### Statistics

Statistical analyses were carried out using SPSS software, version 13.0 (SPSS, Chicago, IL. USA). We applied the Student T-test for invasion assay or the Pearson Chi-Square test to assess the association between p-ERK expression and tumor grade or HGTD-P expression in astrocytic glial tumors. Differences with a *p *value < 0.05 were considered statistically significant.

## Results

### Selection of death-resistant cells by repeated exposure to hypoxia

**Figure 1:**
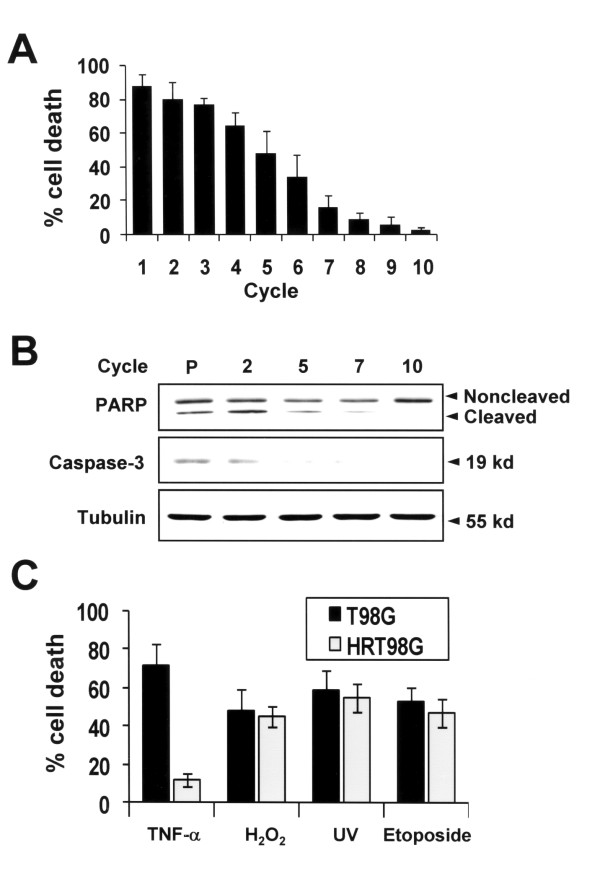
**Selection of HRT98G cells by repeated episodes of hypoxia**. (A) Cell death assay using the trypan blue exclusion method was performed after the indicated cycles of repeated exposure to hypoxia and normoxia. (B) Immunoblot analysis for PARP and cleaved caspase-3 after the indicated cycles. P, parent cells (C) Sensitivity of HRT98G cells to different types of cytotoxic stimuli. T98G and HRT98G cells were exposed to 20 ng TNF-α for 4 h, 100 μM H_2_O_2 _for 24 h, 100 mJ/cm^2 ^UV for 24 h, and 20 μM etoposide for 12 h. Cell death was determined by the trypan blue exclusion method.

To select death-resistant clones of T98G cells induced by hypoxia, we exposed cells to 0.5% O_2 _for 6 h and then returned the cells to normal oxygen tension for recovery. After 6 h of recovery time, detached dead cells were removed and viable cells were further subjected to repeated cycles of hypoxia-normoxia. Cell death rate was determined following the recovery phase of each cycle. As shown in Fig. 1A, more than 85% of cells died after the first cycle, but less than 5% of cells died after 10 repeated cycles. In parallel with cell death rates, caspase-3 and PARP were cleaved after 6 h of hypoxic exposure in parent cells, but not in HRT98G cells (Fig. 1B). Next, to determine whether death resistance of HRT98G cells is specific to hypoxia or not, HRT98G cells were exposed to various damaging stimuli including tumor necrosis factor (TNF)-1α, H_2_O_2_, ultraviolet light (UV), and etoposide. As shown in Fig. 1C, HRT98G was resistant to TNF-1α, but not to H_2_O_2_, UV, and etoposide. Together, our data shows that repeated episodes of hypoxic and normoxic exposure cause T98G cells to survive the low oxygen tension and that the death resistance of HRT98G cells is dependent on the type of injury.

### Alterations of protein expression in death pathways and ROS

**Figure 2:**
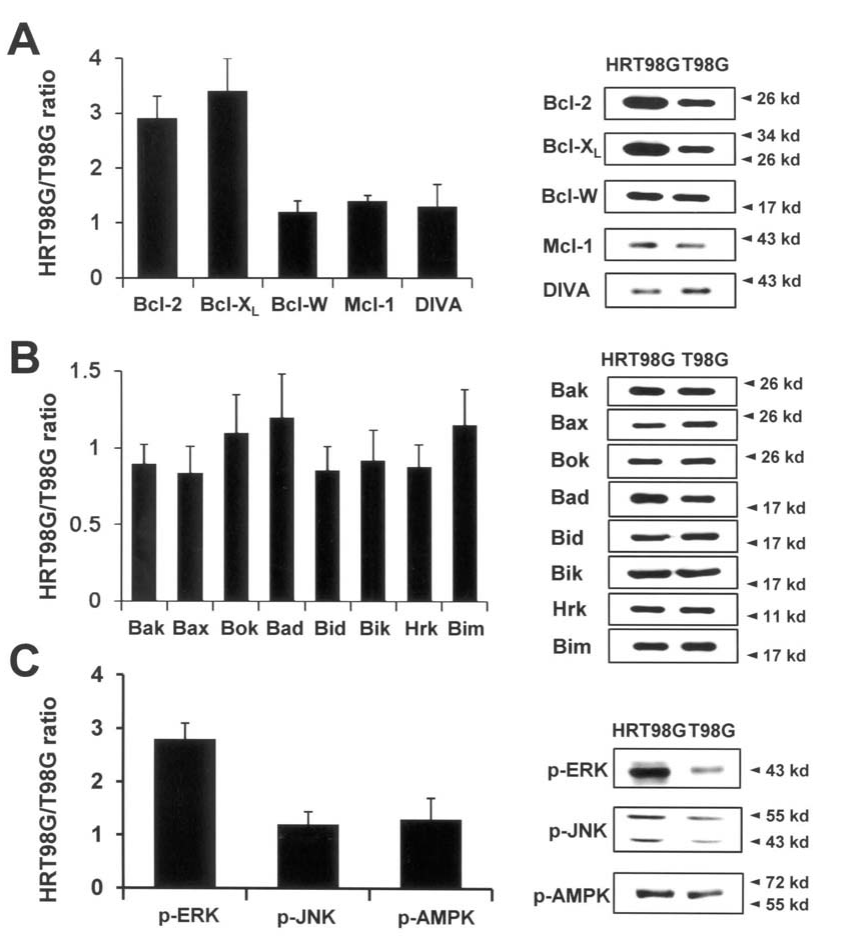
**Difference in protein expression between parent T98G and HRT98G cells**. Proteins were extracted from T98G and HRT98G cells and immunoblots were performed on (A) anti-apoptotic Bcl-2 family proteins, (B) pro-apoptotic Bcl-2 family proteins, and (C) signaling molecules. Densitometric analysis was performed and data is presented as the ratio of protein levels of T98G to HRT98G cells. Representative immunoblots were shown in the right panels of the A, B, and C.

**Figure 3:**
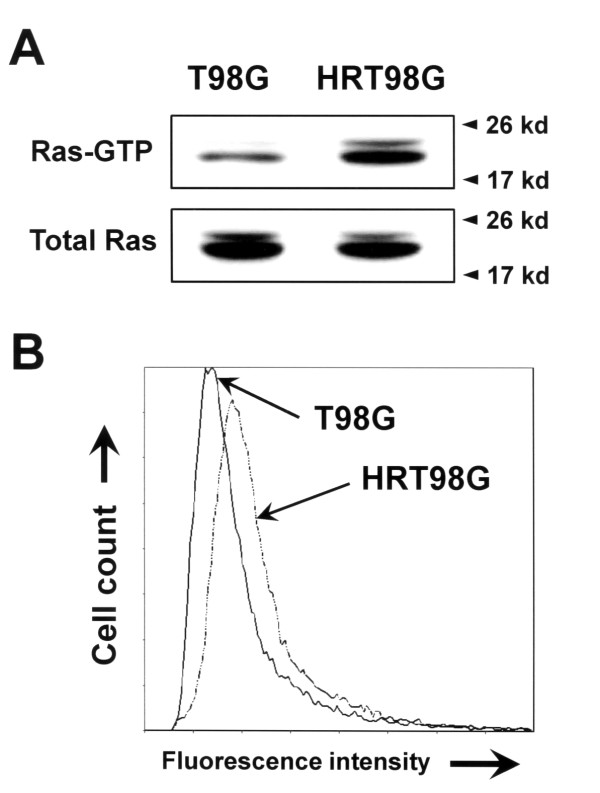
**Determination of Ras activity and ROS level in HRT98G cells**. (A) The Ras activity was assessed by precipitating GTP-bound active Ras with Raf-1 Ras binding domain-agarose conjugate (Top panel). The lower panel demonstrates that equal amounts of Ras were present. (B) The levels of ROS was measured in T98G and HRT98G using DCFH-DA ROS-sensitive fluorescence dye.

To gain insight into the death resistance mechanism of HRT98G cells, we used immunoblot analysis to detect alterations in expression of proteins involved in cell death pathways, such as pro-apoptotic, anti-apoptotic, and signaling proteins. Among the anti-apoptotic proteins, expression of Bcl-2 and Bcl-X_L_, both well-known and common death inhibitor factors, was markedly increased in HRT98G cells compared to parent control cells (Fig. 2A). In contrast, we did not find any significant changes in the expression levels of some other anti-apoptotic proteins, including Bcl-w, Mcl-1, and DIVA (Fig 2A), and pro-apoptotic proteins such as Bak, Bax, Bok, Bad, Bid Bik, Hrk, and Bim (Fig. 2B) between parent cells and HRT98G cells. Next, we examined the signal transducing proteins that transmit death-inducing and death-inhibiting signals, such as ERK, c-jun N-terminal kinase (JNK), and AMP-activated protein kinase (AMPK). Of note, we found that the p-ERK is markedly increased in HRT98G cells compared to parental cells (Fig. 2C). To know the upstream signals responsible for ERK activation in HRT98G cells, we determined Ras activity and ROS level because it has been known that ROS and Ras activation are the initial steps for the activation of MAPK cascades in hypoxic signal transduction [13,14]. As shown in Fig. 3, Ras activity and ROS level were significantly increased in HRT98G cells compared to T98G cells, suggesting that they may be the upstream activators of ERK pathway.

### The upregulation of Bcl-2 and Bcl-X_L _in hypoxia-selected cells is independent of ERK pathway

**Figure 4:**
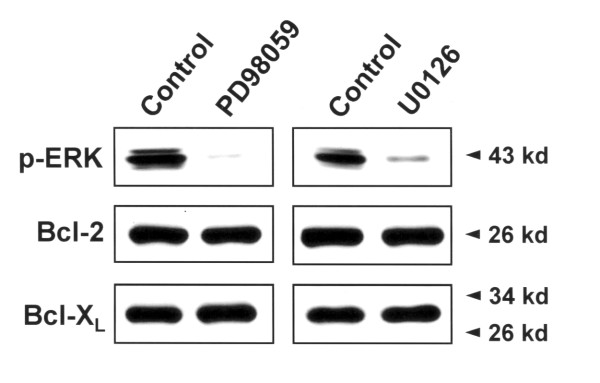
**Effects of ERK on the expression level of Bcl-2 and Bcl-X_L _in HGT98G cells**. HGT98G cells were treated with 30 μM PD98059 (left panel) or 40 μg/ml U0126 (right panel), and the expression levels of Bcl-2 and Bcl-X_L _was determined by immunoblots.

Previously, it has been reported that the ERK activation up-regulates the expression of Bcl-2 and Bcl-X_L_, thereby preventing cell death at the mitochondrial level [15]. Therefore, to examine whether the up-regulation of Bcl-2 and Bcl-X_L _in HRT98G cells was affected by the ERK activation, HRT98G cells were treated with specific ERK inhibitor PD98059 (IC50 ≈7 μM) or U0126 (IC50 ≈0.4 μg/ml), and then the expression levels of Bcl-2 and Bcl-X_L _were determined by immunoblots. As shown in Fig. 4, inhibition of ERK activation did not down-regulate the expressions of Bcl-2 and Bcl-X_L_, suggesting that up-regulation of Bcl-2 and Bcl-X_L _expression by repeated hypoxia did not result from ERK activation.

### Activation of ERK pathways in HRT98G

**Figure 5:**
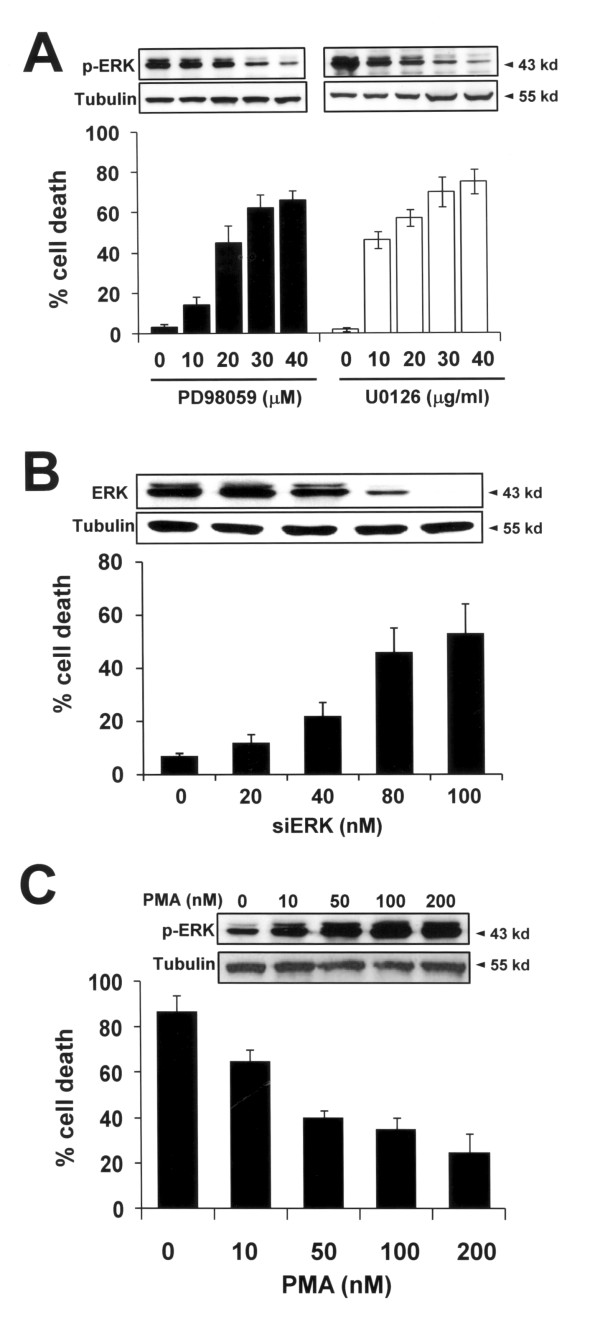
**Activation of ERK in HRT98G cells (A) HRT98G cells were pre-treated with the ERK inhibitor PD98059 or U0126 at the indicated concentration and a trypan blue exclusion assay was performed after 6 h of hypoxia**. Immunoblots for p-ERK and tubulin are shown in the upper panel. (B) HRT98G cells were transfected with the indicated concentration of siERK for 48 h and cell death assay was performed as described in A. Immunoblots for ERK and tubulin are shown in the upper panel. (C) T98G cells were treated with the ERK activator PMA at the indicated concentration and cell death assay was performed as described in A. The immunoblots for p-ERK and tubulin are shown in the upper panel.

Given the higher expression of p-ERK in HRT98G cells, we next investigated whether ERK activation is responsible for the death resistance of these cells. HRT98G cells were treated with PD98059 or U0126, and cells were then subjected to 0.5% hypoxia for 6 h. As shown in Fig. 5A, suppression of ERK activation by these specific inhibitors restored the hypoxia sensitivity of HRT98G cells, suggesting that activation of ERK is a key event responsible for the death resistance of HRT98G cells. The critical role of ERK in hypoxia resistance was reinforced by knockdown of ERK using siRNA (siERK) (Fig. 5B). To confirm our results, we treated T98G cells with the ERK pathway activator PMA [16]. In addition, activation of ERK in T98G cells diminished sensitivity to hypoxia to the level of HRT98G cells (Fig. 3C). Together, our results suggest that ERK activation is mandatory for the hypoxia-induced death resistance of HRT98G cells.

### The effect of ERK activation on *in vitro *invasion

**Figure 6:**
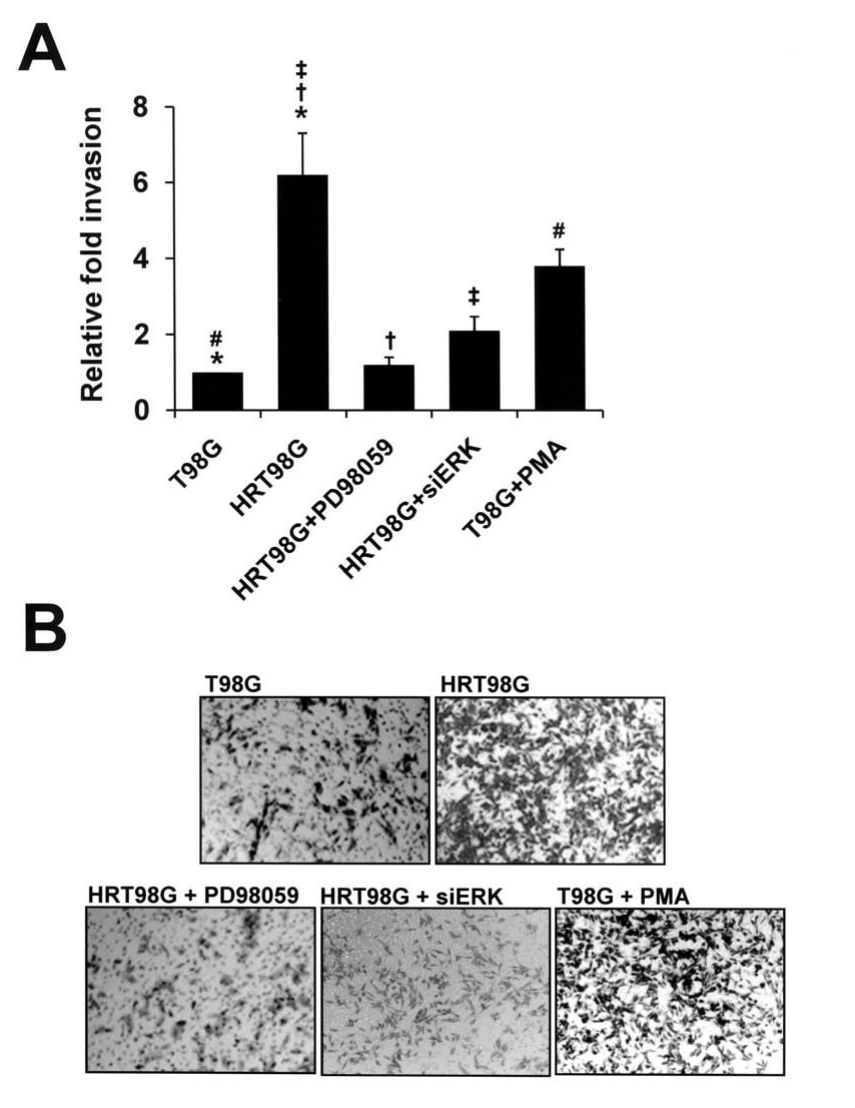
**Activation of ERK facilitates invasive growth of T98G cells**. (A) T98G and HRT98G cells, untreated or treated with 30 μM PD98059, 100 nM siERK, or 100 nM PMA, were plated in Matrigel-coated transwell chambers at 10^4 ^cells per well. After 24 h, the cells on the lower side of the chamber were fixed and stained with hematoxylin. The number of cells that invaded the Matrigel-coated transwell chamber was counted using a light field inverted microscope. The data is the results of three independent experiments in triplicate. An average of six fields of cells was counted under 100× magnification. (*, #, †, ‡, *P *< 0.01). (B) Representative images of the *in vitro *invasion assay.

Invasive growth of tumors is one of the most important hallmarks of malignancy [17,18]. Therefore, we examined whether HRT98G cells are more invasive than their parental cells. Invasion assays were performed using Matrigel-coated transwell invasion chambers, as shown in Fig. 6A, and HRT98G cells were found to be more invasive than their parent cells. To determine if ERK activation was responsible for the invasive phenotype of HRT98G cells, we performed invasion assays with T98G and HRT98G cells, untreated or treated with PD98059, siERK, or PMA. Inactivation of ERK by PD98059 or siERK suppressed the invasive potential of HRT98G cells (Fig. 6A). In contrast, increased invasiveness was observed in PMA-treated ERK-activated T98G cells (Fig. 6A). Representative images of the invasion assay are shown in Fig. 6B. This result suggests that activation of the ERK pathway is a key event responsible for the aggressive behavior of HRT98G cells.

### Activation of ERK in hypoxic tumor tissue

**Figure 7:**
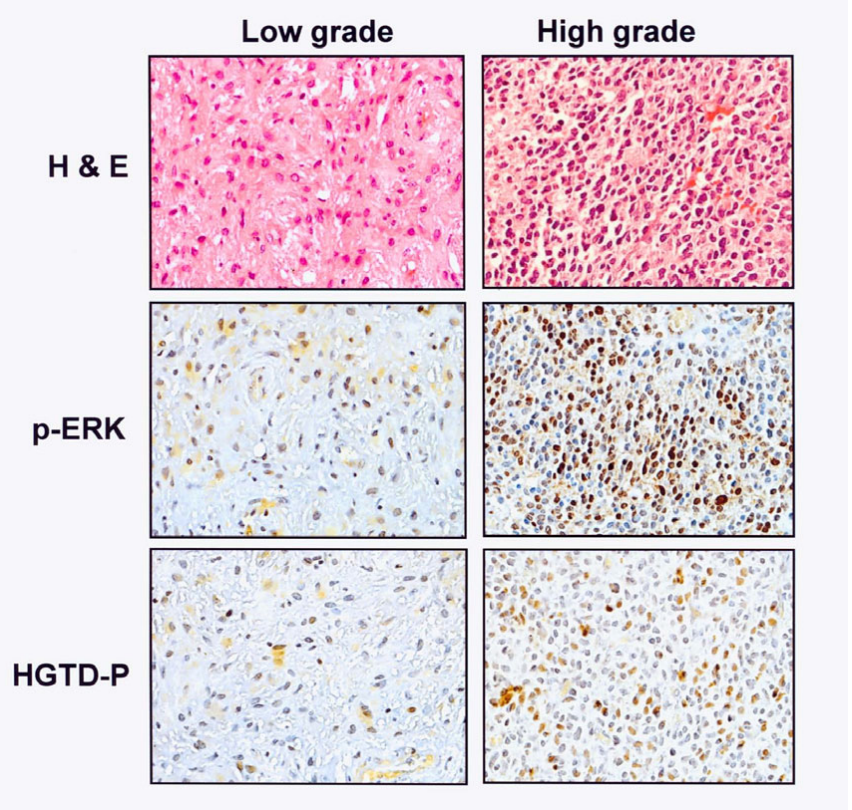
**Representative hematoxylin and eosin (H&E) and immunohistochemical pictures of p-ERK and HGTD-P in low (grade II)- and high (grade IV)- grade astrocytic glial tumors**.

To confirm that ERK activation is important for the aggressive behavior induced by chronic hypoxia, we examined whether ERK is activated in the region of the tumor exposed to hypoxia. To this end, 10 low- and high-grade astrocytic glial tumors were immunohistochemically stained for p-ERK and HGTD-P, marker proteins expressed in hypoxia [11]. The expression of p-ERK was correlated with tumor grade (Table 1). All high grade tumors (grade III and IV) showed 3+ or 4+ expression of p-ERK protein, whereas 70% of low grade tumors (grade I and II) exhibited 1+ or 2+ expression of the protein (*p *= 0.006). The results for p-ERK expression were similar to those for HGTD-P expression with statistical significance (*p *= 0.027) (Table 2). Representative immunohistochemical staining is shown in Fig. 7. These results suggest that the ERK pathway is activated in tumor areas exposed to hypoxia.

**Table 1:** Comparison of p-ERK expression with tumor grade in astrocytic glial tumors

WHO grade			Expression of p-ERK (%)			
			
			1+	2+	3+	4+
**Low grade**	**Grade I**	**(n = 7)**	2 (28)	3 (43)	2 (28)	0 (0)
	**Grade II**	**(n = 3)**	0 (25)	2 (50)	1 (25)	0 (0)
**High grade**	**Grade III**	**(n = 3)**	0 (0)	0 (0)	2 (67)	1 (33)
	**Grade IV**	**(n = 7)**	0 (0)	0 (0)	3 (43)	4 (57)

*p *= 0.006, analyzed by Pearson Chi-Square test.

**Table 2:** Comparison of p-ERK expression with HGTD-P expression in astrocytic glial tumors

Protein		Expression of p-ERK (%)			
	
	Immunoreactivity	1+ (n = 2)	2+ (n = 5)	3+ (n = 8)	4+ (n = 5)
**HGTD-P**	1+ (n = 3)	0 (0)	3 (100)	0 (0)	0 (0)
	2+ (n = 7)	2 (28.6)	1 (14.3)	4 (57.1)	0 (0)
	3+ (n = 6)	0 (0)	1 (16.7)	2 (33.3)	3 (50.0)
	4+ (n = 4)	0 (0)	0 (0)	2 (50.0)	2 (50.0)

*p *= 0.027, analyzed by Pearson Chi-Square test.

## Discussion

The chronic hypoxia that results from the rapid growth of tumor parenchyma can select for cancer cells with a more aggressive behavior and a death-resistant phenotype. Thus, identifying the key molecules responsible for these phenotypic changes and their molecular mechanisms is important to develop effective therapeutic modalities. The aim of this study was to identify the molecular basis of the phenotypic changes triggered by chronic repeated hypoxia. Initially, we selected death-resistant clones from T98G cells after repeated episodes of hypoxia and normoxia. Over 95% of HRT98G cells selected by more than 10 hypoxic cycles survived after 6 hours of hypoxia. However, death resistance was not specific for hypoxia only because cells were also resistant to TNF-α-induced death. This suggests that death resistance induced by repeated hypoxic cycles results from a common downstream death pathway. While up-regulation of death-inhibitory proteins like Bcl-2 and Bcl-X_L _in HRT98G cells may support this hypothesis, different sensitivities to other types of injuries such as H_2_O_2 _and UV indicate that death resistance cannot be solely explained by anti-apoptotic Bcl-2 family proteins. Previously, Dong and Wang [19] found that immortalized rat kidney epithelial cells selected by hypoxia were cross-resistant to the diverse apoptotic stimuli such as staurosporin, azide, and cis-platin, at the mitochondrial level by up-regulation of Bcl-X_L_, but not by Bcl-2. Although this discrepancy in Bcl-2 expression involved in death-resistant phenotypic changes may come from the difference in cell types and cytotoxic stimuli used, further studies are required for the explanation of the precise molecular mechanisms. Nevertheless, our study on the gene expression in hypoxia-selected cells showed that ERK activation is crucial for the invasive potentials of selected cells as well as death resistance.

ERK is activated by MAPK in response to growth stimuli and involved in diverse cellular signaling pathways, including pathways involved in survival and proliferation [20,21]. Our results show that repeated exposure to hypoxia-normoxia results in activated ERK pathways through Ras activation. While there is accumulating evidence that ERK activation induces cell proliferation and inhibition of apoptosis, the signaling mechanism underlying ERK activation by repeated hypoxia-reoxygenation is not clear. If the hypoxia-reoxygenation cycles are considered to be a type of oxidative stress on the cells, then ERK might be activated via reactive oxygen species (ROS)-dependent pathways, as shown in Fig 3. NADPH oxidase activation and subsequent ROS generation shortly after reoxygenation have been suggested to initiate ERK signaling [22,23]. In support of this, Wang et. al. [24] and Kumar et. al. [25] have reported that hypoxia/reoxygenation or oxidative stress induces ERK activation, which is required for the aggressive phenotype of prostatic cancer.

Astrocytic glial tumors, including low-grade (WHO grade I and II) and high-grade astrocytoma (WHO grade III and IV), are the most common tumors of glial cell origin [26,27]. In our study, immunohistochemical staining of astrocytic glial tumors showed that tumor cells with aggressive and invasive behavior express higher levels of p-ERK and HGTD-P than low-grade tumor tissues. These data confirm the role of p-ERK in the chronic hypoxic-induced aggressive cell phenotype.

We have shown that ERK activation influences the development of an invasive phenotype. The generality of increased invasiveness mediated by ERK activation is an important question. In our system, we found that the amount of matrix metalloproteinase-3 (MMP3) transcript was significantly increased in HRT98G cells (data not shown). MMP3 up-regulation by activated ERK may be responsible for the invasiveness of HRT98G cells. In future studies, we intend to investigate the role of signaling pathways downstream of ERK in hypoxic cell death resistance.

## Conclusion

Collectively, we showed that repeated episodes of exposure to hypoxia and normoxia change T98G cells to HRT98G cells that have a more death-resistant and invasive phenotype. As compared with parent cells, HRT98G cells express higher levels of anti-apoptotic proteins such as bcl-2, Bcl-X_L_, and p-ERK. Activation or suppression of ERK pathways with a specific activator or inhibitor, respectively, demonstrates that ERK is a key molecule responsible for the death resistance associated with hypoxia and a more invasive phenotype. Finally, we show that the activation of ERK is more prominent in high grade astrocytic glial tumors exposed to hypoxia than in low grade tumors. Our results may be helpful in developing appropriate and effective cancer treatment modalities.

## Abbreviations

AMPK: AMP-activated protein kinase; EMT: Epithelial-Mesenchymal Transition; ERK: Extracellular signal-Regulated Kinase; Glut-1: Glucose transporter protein-1; HIF-1: Hypoxia Inducible Factor-1; JNK: c-Jun N-terminal Kinase; MAPK: Matogen-Activated Proein Kinase; MMP3: Matrix Metalloproteinase-3; PARP: Poly (ADP-ribose) polymerase; PKC: Protein Kinase C; PMA: Phorbol Myristate Acetate; ROS: Reactive Oxygen Species; TNF: Tumor Necrosis Factor; UV: Ultraviolet; VEGF: Vescular Endothelial Growth Factor;

## Competing interests

The authors declare that they have no competing interests.

## Authors' contributions

JYK conceived of the study and its design, performed the experiments, and helped to draft the manuscript. YJK carried out immunoblot analysis and cell death assay. SL provided glial tumor tissues, analyzed clinical data, and performed statistical analysis. JHP provided overall study design, guidance, and drafted the manuscript. All authors read and approved the final manuscript.

## Pre-publication history

The pre-publication history for this paper can be accessed here:

http://www.biomedcentral.com/1471-2407/9/27/prepub
